# Phase-I trial of oral fluoropyrimidine anticancer agent (S-1) with concurrent radiotherapy in patients with unresectable pancreatic cancer

**DOI:** 10.1038/sj.bjc.6603735

**Published:** 2007-04-17

**Authors:** H Shinchi, K Maemura, H Noma, Y Mataki, T Aikou, S Takao

**Affiliations:** 1 Department of Surgical Oncology and Digestive Surgery, Kagoshima University, Kagoshima, Japan; 2 Frontier Science Research Center, Kagoshima University, Kagoshima, Japan

**Keywords:** phase-I study, S-1, pancreatic cancer, chemoradiation

## Abstract

In this phase-I trial, we evaluated the safety of S-1, a novel oral fluoropyrimidine anticancer agent, combined with external-beam radiotherapy (EBRT) to determine the maximum-tolerated dose and dose-limiting toxicity (DLT) in unresectable pancreatic cancer patients. Patients had histologically proven unresectable locally advanced or metastatic pancreatic cancer. S-1 was administered orally twice daily. External-beam radiotherapy was delivered in fractions of 1.25 Gy × 2 per day, totalling 50 Gy per 40 fractions for 4 weeks. S-1 was given at five dose levels: 60 mg m^–2^ day^–1^ on days 1–7 and 15–21 (level 1), 1–14 (level 2), and 1–21 (level 3a) and 80 mg m^–2^ day^–1^ on days 1–21 (level 3b) and 1–28 (level 4). We studied 17 patients: dose levels 1 (four patients), 2 (four patients), 3a (three patients), 3b (three patients), and 4 (three patients). One patient in level 1 (grade 3 vomiting) and two patients in level 4 (grade 4 neutropenia and grade 3 anorexia) showed DLT. No DLT was seen for levels 2, 3a, and 3b. Clinical effects by computed tomography included 5 partial responses (35%), 11 cases of stable disease, and one case of progressive disease. CA19–9 levels of less than half the starting values were observed in 8 of 16 (50%) patients. S-1 at a dose of 80 mg m^–2^ day^–1^ given on days 1–21 is safe and recommended for phase-II study in patients with locally advanced and unresectable pancreatic cancer when given with EBRT.

Adenocarcinoma of the exocrine pancreas (pancreatic cancer) is a morbid disease with a dismal prognosis. At present, complete surgical resection offers the best chance of cure (Yeo *et al*, 1995; Takao *et al*, 1999). However, because of the invasion of major vessels or the presence of metastasis, most pancreatic cancers are already unresectable at the time of diagnosis (Niederhuber *et al*, 1995). Without surgical intervention, the median survival is less than 6 months (Matsuno *et al*, 2004). Consequently, many studies have recommended radiation therapy or chemotherapy as the primary treatment for patients with unresectable pancreatic cancers. For patients with locally unresectable disease, the results of previous randomised trials by the Gastrointestinal Tumour Study Group indicate that concurrent external-beam radiation therapy (EBRT) and 5-fluorouracil (5-FU) therapy results in significantly better survival than EBRT or chemotherapy alone (Gastrointestinal Tumour Study Group, 1981, 1988). Since then, concurrent EBRT and 5-FU have been generally accepted as the standard treatment for locally advanced pancreatic cancer (Klaassen *et al*, 1985; Poen *et al*, 1999; Paulino and Latona, 2000; Shinchi *et al*, 2002). The benefit seen in the early combined-modality trials was modest, however, with a median survival of only 10 months. To improve the efficacy of the treatment, various anticancer agents such as gemcitabine and radiation schedules are being examined in clinical trials (Burris *et al*, 1997; Blackstock *et al*, 1999; McGinn *et al*, 2001; de Lange *et al*, 2002; McGinn and Zalupski, 2003; Okusaka *et al*, 2004). As yet, however, no regimen has been shown to be superior to conventional chemoradiotherapy with 5-FU. Thus, there is a need to develop new agents and combination regimens to achieve greater survival benefit in patients with unresectable advanced pancreatic cancer.

S-1 is a new oral fluoropyrimidine derivative consisting of tegafur and two modulators, 5-chloro-2,4-dihydroxypyridine (CDHP) and potassium oxonate in a molar ratio of 1 : 0.4 : 1 (Shirasaka *et al*, 1996). 5-Chloro-2,4-dihydroxypyridine is a reversible competitive inhibitor of dihydropyrimidine dehydrogenase, which is an enzyme for 5-FU degradation. Therefore, CDHP with tegafur is expected to yield prolonged 5-FU concentrations in serum and tumour tissue. Potassium oxonate is a reversible competitive inhibitor of orotate phosphoribosyltransferase, which is an enzyme for 5-FU phosphoribosylation in the gastrointestinal mucosa. It is reported that potassium oxonate ameliorates the gastrointestinal toxicity of tegafur by decreasing 5-fluorodeoxyuridine monophosphate production in the gastrointestinal mucosa (Shirasaka *et al*, 1993). Recent clinical trials of S-1 have shown promising results in various solid tumours (Koizumi *et al*, 2000; Ohtsu *et al*, 2000; Kawahara *et al*, 2001). In an early phase-II clinical study for metastatic pancreatic cancer in Japan, the objective response rate of S-1 as a single agent was 21.1% (4/19) (Ueno *et al*, 2005).

S-1 has also been shown to be a potent radiosensitizer in human solid tumour xenograft (Fukushima *et al*, 1998; Harada *et al*, 2004), which suggests that the combination of radiotherapy and S-1 may improve survival in patients with locally advanced pancreatic cancer. However, no previous reports have described the efficacy and safety of chemoradiation therapy with S-1 for the treatment of pancreatic cancer. Thus, we conducted a phase-I study to determine the maximum-tolerated dose (MTD) and dose-limiting toxicity (DLT) of S-1 and radiation combination therapy in patients with unresectable pancreatic cancer.

## PATIENTS AND METHODS

### Eligibility

Patients with histologically or cytologically confirmed adenocarcinoma of the pancreas were enrolled onto this study from July 2004 to December 2005 at the Kagoshima University Hospital. Eligible patients were clinically or surgically staged and considered unresectable for cure. Patients with metastatic disease were eligible if the principal symptom associated with their primary disease was pain and if they had an estimated life expectancy greater than 6 months. Eligibility criteria also included the following: age ⩾20 years; Eastern Cooperative Oncology Group performance status of 0–2; measurable or assessable disease; life expectancy of >3 months; adequate organ function defined as granulocyte count of ⩾1500 *μ*l^–1^, haemoglobin ⩾8 g dl^–1^, platelet count ⩾100 000 *μ*l^–1^, bilirubin ⩽1.5 mg dl^–1^, and creatinine ⩽2.5 mg dl^–1^. If the patients had any previous chemotherapy, radiotherapy or combined-modality treatment for either advanced disease or postoperative adjuvant therapy, that treatment had to have been discontinued for at least 4 weeks before entry into the study.

The exclusion criteria were as follows: active infection, severe heart disease, interstitial pneumonitis or pulmonary fibrosis, pleural effusion or ascites, active gastroduodenal ulcer, pregnant or lactating females, severe mental disorder, active concomitant malignancy, and other serious medical conditions. The patients who did not have sufficient integrity of the gastrointestinal tract or who had malabsorption syndrome were also excluded. The protocol was approved by the Human Studies Group at the Kagoshima University School of Medicine. All patients gave written informed consent before participation.

### Treatment program

S-1 was supplied by Taiho Pharmaceutical Co. Ltd (Tokyo, Japan). Each S-1 capsule contained 20 or 25 mg of tegafur. Individual doses were rounded as closely as possible to the calculated dose, given the available formulation. The study drug was administered orally in two divided doses after breakfast and supper at a dosage of 60 or 80 mg m^–2^ day^–1^. Patients were scheduled to receive S-1 at five levels (1, 2, 3a, 3b, and 4), as listed in Table 1.

External-beam radiotherapy was delivered with 10 MV photons by using a conformal technique in fractions of 1.25 Gy × 2 per day, 5 days per week, totaling 50 Gy per 40 fractions for 4 weeks. The radiation field included the primary tumour and adjacent lymph nodes (pancreaticoduodenal and celiac axis), as defined by treatment-planning computed tomography (CTS-20 SPS, Shimadzu Co. Kyoto, Japan) performed 1 or 2 days before treatment.

### Toxicity and efficacy evaluation

Toxicity was graded according to the National Cancer Institute: Common Toxicity Criteria version 2.0. The DLT was reached when any of the following occurred: grade 3 leukocytopaenia and/or neutropaenia with high fever, grade 4 haematologic toxicities, or grade 3 or 4 nonhaematologic toxicity excluding nausea, vomiting, anorexia and fatigue in the absence of appropriate antiemetics, or grade 3 or 4 nausea/vomiting uncontrolled by aggressive antiemetic support, or delay of recovery from treatment-related toxicity for more than 2 weeks.

Standard antiemetic therapy was prescribed as required. Antidiarrhoeal drugs were not given prophylactically but could be used for the symptomatic treatment of diarrhoea of grade 2 or higher. Chemotherapy was withheld upon development of a grade 2 or higher nonhaematologic toxicity or grade 3 or higher haematologic toxicity, and resumed at the same dose level when symptoms were grade 1 or when granulocyte and platelet counts were 1500 and 100 000 ml^−1^, respectively. Radiation could be held for toxicity at the discretion of the treating physician.

At least three patients were enrolled at each dose level. If DLT was observed in the initial three patients, a maximum of three additional patients was entered into the same dose level. The MTD was defined as the dose at which two or more patients developed DLT.

Physical examinations, complete blood cell counts, and biochemistry tests were performed at least once weekly. Tumour assessment by computed tomography was performed every 3 months. Tumour responses were evaluated according to the World Health Organization's criteria (World Health Organization, 1980). Serum CA19-9 concentrations were measured every 4 weeks. A value of 37 U ml^–1^ was defined as the upper limit of normal. Overall survival time was calculated from the date of treatment initiation to the date of death or the last follow-up. Progression-free survival time was calculated from the date of treatment initiation until documented disease progression or death due to any cause (whichever occurred first).

## RESULTS

### Patient characteristics

Between August 2004 and December 2005, 17 patients were enrolled in the study: 10 men and seven women. The median age of the participants was 66 years (range, 40–79 years). Patient characteristics are summarised in Table 2. Eleven patients had locally advanced unresectable disease without distant metastases, and six patients had metastatic disease. Before the start of the study, two patients had undergone gastrojejunostomy for duodenal obstruction, and nine had undergone biliary drainage for obstructive jaundice. Ten patients had abdominal or back pain at study entry. Patients were treated with S-1 and concurrent radiation as listed in Table 1. The first patient developed DLT at level 1. Next, three patients were entered at the same level. With no further DLT demonstrated in these three consecutive patients, level escalation proceeded. Four patients were initially enrolled at level 2 and no DLT was observed at this level. As a result, a total of four patients were treated at both level 1 and level 2.

### Toxicity and recommended dose

All 17 patients completed the combined S-1 and radiation therapy and were evaluated for toxicity, as listed in Table 3. No treatment-related deaths occurred during the study. Haematologic toxicity, particularly leukopaenia (⩾grade 1:35%), was a common effect of combined S-1 and radiation therapy with this schedule of administration. Gastrointestinal toxicity, such as anorexia (⩾grade 1:53%) and nausea (⩾grade 1:35%), was also frequently seen, but was usually mild and transient.

Three patients showed signs of DLT: one patient developed grade 3 vomiting at the level 1 dose and one developed grade 3 anorexia at the level 4 dose. These patients quickly recovered with 7 days rest of chemoradiation. In addition, one patient developed grade 4 neutropaenia at the level 4 dose; this was observed during the last 5 days of treatment and resolved shortly after the completion of treatment. No DLT was observed at the 2, 3a, and 3b dose levels (Table 3). Because two of three patients receiving the level 4 dose showed DLT, level 4 was considered the MTD, and level 3b (80 mg m^–2^ day^–1^ given on days 1–21) is proposed as the recommended dose level for further studies with this schedule.

### Efficacy

The objective tumour responses at each dose level are shown in Table 4. Of the 17 enrolled patients, six patients (36%) had a PR, 10 (59%) had radiologically SD, and the other one patient (5%) had PD. The serum CA19-9 concentration was reduced to less than 50% of baseline values in 8 (50%) of 16 patients who had a pretreatment value of more than the upper limit of normal (37 U ml^–1^). At the time of analysis, 14 patients died because of disease progression. The median overall survival was 12.3 months (range: 2.7–18.2 months) and the median disease-free survival was 7.0 months (range: 1.2–14.5 months).

## DISCUSSION

Concomitant radiotherapy and chemotherapy is commonly used to treat locally unresectable pancreatic cancers (Moertel *et al*, 1969; Gastrointestinal Tumour Study Group, 1981, 1988; Klaassen *et al*, 1985; Poen *et al*, 1999). 5-fluorouracil has been used for chemoradiation because it can enhance the effects of radiation (Whittington *et al*, 1995; Ishii *et al*, 1997). The novel oral anticancer agent S-1 was developed to improve the tumor-selective toxicity of 5-FU and has shown a good efficacy for a variety of solid tumours, including pancreatic cancer (Koizumi *et al*, 2000; Ohtsu *et al*, 2000; Kawahara *et al*, 2001; Ueno *et al*, 2005). Thus, S-1 may also act as a radiosensitizer because it has cytotoxic mechanisms similar to those of 5-FU (Hirata *et al*, 1999; Hoff *et al*, 2003; Schoffski, 2004). Harada *et al* (2004) evaluated S-1 in combination with ionising radiation both *in vivo* and *in vitro* against human oral cancer cell lines. They showed that the combination of S-1 and radiation was more effective than either agent alone, and that S-1 administration before radiation was more effective than administration after radiation. As stated above, S-1 has also been shown to be a potent radiosensitizer, which suggests that the combination of radiotherapy and S-1 may improve survival in patients with locally advanced pancreatic cancer. Thus, we performed a phase-I study to evaluate the efficacy and safety of combined S-1 and radiation therapy in patients with unresectable pancreatic cancer.

S-1 has already undergone phase I and phase II testing in several solid tumours in Japan and Western countries (Koizumi *et al*, 2000; Ohtsu *et al*, 2000; van Groeningen *et al*, 2000; Kawahara *et al*, 2001; Hoff *et al*, 2003; Ueno *et al*, 2005; Nakamura *et al*, 2006). The main adverse reaction was myelosuppression in a Japanese phase-I study, and diarrhoea in a European and a North-American phase-I study (van Groeningen *et al*, 2000; Hoff *et al*, 2003). It is well known that total dose tolerated in Western populations when used in the advanced settings is significantly less tolerated than in Eastern populations. van Groeningen *et al* (2000) have reported that this might be due to the polymorphism of cytochrome *P*450, with differences in activity among patients with different ethnic backgrounds. They have also reported that cytochrome *P*450 seemed to be involved in the conversion of tegafur into 5-FU, resulting in higher 5-FU levels in Western populations (van Groeningen *et al*, 2000). In Japan, the standard single-agent dose is 80 mg m^–2^ day^–1^ twice daily for 28 consecutive days, followed by 14 days of rest, although the RD of S-1 was 70–80 mg m^–2^ in Europe, and 60 mg m^–2^ in the US. In the present phase-I study, we have examined the appropriate dose and duration of S-1 and the safety of S-1 with radiation therapy. The dosage was escalated from 60 mg m^–2^ day^–1^ at level 1–3a to 80 mg m^–2^ day^–1^ at levels 3b and 4, respectively. In addition, S-1 was administered for 14 days in levels 1 and 2, for 21 consecutive days in level 3, and for 28 consecutive days in level 4. Dose-limiting toxicity was observed in two of three patients at level 4, which was defined as the MTD. No unexpected or life-threatening toxicities were observed during the study. Consequently, the recommended dose of S-1 combined with radiation was the level 3b dose: 80 mg m^–2^ day^–1^ given on days 1–21.

The combination of S-1 and radiation showed a good objective response rate of 35% (6/17), with a good tumour growth control rate (PR plus SD) of 94% (16/17). Two (67%) of three patients at level 3b, which was considered the recommended dose, obtained PR, which suggests promising antitumour efficacy at this dose schedule. Moreover, the serum CA19-9 level was reduced more than 50% in 8 (50%) of 16 patients with an abnormal pretreatment level. These results are encouraging and comparable to those of patients treated with chemoradiation at other treatment schedules (Gastrointestinal Tumour Study Group, 1981, 1988; Klaassen *et al*, 1985; Blackstock *et al*, 1999; McGinn *et al*, 2001; de Lange *et al*, 2002; Shinchi *et al*, 2002; McGinn and Zalupski, 2003; Okusaka *et al*, 2004).

In patients with locally advanced pancreatic cancer treated with chemoradiation it is important to enhance local tumour control and simultaneously reduce the risk of distant metastases. S-1 not only has an ability of local disease control as a potent radiosensitizer but also has systemic effects as a chemotherapeutic agent (Schoffski, 2004). Thus, S-1 with radiation may result in improved long-term survival in chemoradiation treated patients. Moreover, because S-1 is administered orally, this combination therapy is quite feasible in the outpatient treatment setting without hospitalisation. The ability of S-1 to deliver protracted plasma concentrations of 5-FU without the need for intravenous access or an infusion pump makes it an attractive alternative in regimens combining chemotherapy and radiation.

In conclusion, our combination regimen of S-1 and radiation is a promising and well-tolerated approach with possible application at an outpatient clinic. S-1 at a dosage of 80 mg m^–2^ day^–1^ given on days 1–21 is safe and recommended for further studies when given with EBRT. Phase-II study of this regimen is underway in patients with locally advanced and unresectable pancreatic cancer.

## Figures and Tables

**Table 1:** S-1 Dose levels and schedule

**Dose level**	***n***	**S-1 dose (mg m^−2^)**	**S-1 schedule**
1	4	60	Days 1–7, 15–21
2	4	60	Days 1–14
3a	3	60	Days 1–21
3b	3	80	Days 1–21
4	3	80	Days 1–28

**Table 2:** Patient characteristics

**Characteristics**	**No. of patients (%)**
Patients enrolled	17
	
*Gender*	
Men	10 (59)
Women	7 (41)
	
*Age (years)*	
Median (range)	66 (40–79)
	
*ECOG performance status*	
0	8 (47)
1	7 (41)
2	2 (12)
	
*Tumour location*	
Head	12 (71)
Body tail	5 (29)
	
*Stage of disease*	
Locally advanced alone	11 (65)
Locally advanced and metastatic	6 (35)
	
*Sites of metastatic disease*	
Liver	1 (6)
Distant lymph nodes	5 (29)

ECOG=Eastern Cooperative Oncology Group.

**Table 3:** Grade 3 or higher toxicities

	**Level**	**1 (*n*=4)**		**2 (*n*=4)**		**3a (*n*=3)**		**3b (*n*=3)**		**4 (*n*=3)**	
**Toxicity**	**grade^a^**	**3**	**4**	**3**	**4**	**3**	**4**	**3**	**4**	**3**	**4**
Neutropaenia		0	0	0	0	0	0	0	0	0	1
Vomiting		1	0	0	0	0	0	0	0	0	0
Anorexia		0	0	0	0	0	0	0	0	1	0

a CI=Common Toxicity Criteria, version 2.0.

**Table 4:** Objective tumour response

		**Response**				
**Dose level**	***n***	**CR**	**PR**	**s.d.**	**PD**	**Response rate (%)**
1	4	0	1	2	1	25
2	4	0	1	3	0	25
3a	3	0	1	2	0	33
3b	3	0	2	1	0	67
4	3	0	1	2	0	33
Total	17	0	6	10	1	35
